# Clinico-pathological features of tuberculosis due to *Mycobacterium tuberculosis* Uganda genotype in patients with tuberculous lymphadenitis: a cross sectional study

**DOI:** 10.1186/1472-6890-14-14

**Published:** 2014-04-02

**Authors:** Dan Wamala, Benon Asiimwe, Edgar Kigozi, Gerald Mboowa, Moses Joloba, Gunilla Kallenius

**Affiliations:** 1 Department of Pathology, Mulago Hospital and Makerere University College of Health Sciences, PO Box 7072, Kampala, Uganda; 2 Department of Medical Micobiology, Makerere University College of Health Sciences, PO Box 7072, Kampala, Uganda; 3 Department of Clinical Sciences and Education, Sodersjukhuset, Karolinska Institute, SE-171 77 Stockholm, Sweden

**Keywords:** Tuberculosis, Extra pulmonary, Mycobacterium, Genotype, Abdominal lymphadenopathy, Uganda

## Abstract

**Background:**

Tuberculous lymphadenitis is next to pulmonary tuberculosis as the most common cause of tuberculosis. Uganda genotype, one of the sub-lineages of *Mycobacterium tuberculosis*, is the most prevalent cause of pulmonary tuberculosis in Uganda. We here investigate the clinicopathological characteristics of patients with tuberculous lymphadenitis infected with *M. tuberculosis* Uganda genotype compared with those infected with *M. tuberculosis* non-Uganda genotype strains.

**Methods:**

Between 2010 and 2012, we enrolled 121 patients (mean age 28.5 yrs, male 48%; female 52%) with tuberculous lymphadenitis, and categorized them by their *M. tuberculosis* genotypes. The clinical features and lymph node cytopathological parameters were compared between patients in the Uganda and non-Uganda categories using a crude and multivariable logistic regression model with adjustment for confounding factors.

**Results:**

Of the 121participants, 56 (46%) were infected with strains of Uganda genotype. Patients infected with this genotype had significantly lower frequency of abdominal lymphadenopathy (odds ratio 0.4, p = 0.046) after adjusting for sex, age and HIV. Abdominal lymphadenopathy was also significantly associated with abnormal chest X-ray (p = 0.027).

**Conclusion:**

Tuberculous lymphadenitis patients infected with *M. tuberculosis* Uganda genotype were significantly less prone to have abdominal lymphadenopathy indicating potential reduced ability to disseminate and supporting the concept that differences in *M. tuberculosis* genotype may have clinical implications.

## Background

Tuberculosis (TB) remains a major global health problem. The latest estimates by WHO reveal that there were almost 8.6 million new cases in 2012 and 1.3 million TB deaths, 980 000 among HIV negative people and 320 000 HIV-associated TB deaths [1]. Uganda is ranked 16^th^ among the 22 high burden countries with prevalence of 175 cases per 100,000 in 2012 of all forms of TB [2], and 60% of the patients are co-infected with HIV. The HIV prevalence in the Ugandan adult population is 6.7% [3].

Various host determinants such as sex, age and HIV co-infection influence the development of TB [4,5], and the clinical outcome of *Mycobacterium tuberculosis* infection has been mainly attributed to host factors [6-9]. However, there is a broad variation in clinical pathological manifestations of TB in both HIV positive and HIV negative patients, which cannot be explained exclusively by host or environmental factors. Autopsy observations in Mulago Hospital, Kampala, have revealed severe or disseminated TB in HIV negative cases (unpublished information).

*M. tuberculosis* has evolved into a number of widely distributed and genetically diverse lineages and sub-lineages [10] exhibiting variable disease phenotypic characteristics demonstrated in epidemiological studies and animal models [11,12]. This together with diversity of the host immunological response may explain the wide clinical-immunopathological spectrum of disease in those patients who cannot control the infection [13].

*M. tuberculosis* Uganda genotype causes up to 70% of pulmonary TB in Kampala [14], and other districts of Uganda [15,16]. The Uganda genotype as cause of other disease manifestations has however not been studied. Although TB is mainly a pulmonary disease, *M. tuberculosis* can infect any part of the body. Extrapulmonary TB (EPTB) has been reported in 15–20% of HIV-negative patients [17,18], and the proportion is much higher in patients infected with HIV [17-19]. EPTB is thought to be caused by spread of bacteria by the haematogenous route from an initial focus in the lung and as such to represent a disseminated form of TB disease [20]. The commonest manifestation of EPTB is in most settings peripheral lymphadenitis [19,21,22].

The aim of this study was to assess the clinico-pathological features of TB in patients with peripheral TB lymphadenitis and to determine whether those infected with strains of Uganda genotype display specific clinical, radiological and cytopathological features compared to those infected with other genotypes of *M. tuberculosis*.

## Methods

### Ethical considerations

Ethical approval for the study was granted by the Institutional Review Board of Makerere University School of Medicine and the Uganda National Council for Science and Technology. Written informed consent to obtain samples as well as to use isolates from the samples for studies was obtained from all enrolled study participants or their legal guardians.

### Study population

This was a cross sectional study conducted between February 2010 and July 2012, at the fine needle aspiration clinic, Mulago Teaching and National Referral Hospital, Kampala and Department of Medical Microbiology, Makerere University College of Health Sciences. Patients with persistent lymphadenopathy at this hospital are routinely referred from medical or surgical units. In this study, 283 ethnic African patients with superficial lymphadenopathy and evening fever were enrolled.

Patients presenting with superficial persistent lymph node enlargement, and cytological and clinical features of TB were recruited into the study after written informed consent. The clinical features of TB included unintentional weight loss, fatigue, night sweats, evening fevers for more than 4 weeks and loss of appetite. Tuberculous lymphadenitis was defined based on: i) superficial lymphadenopathy equal to or greater than 1x1cm persistent for more than 4 weeks. ii) cytological features consistent with TB and iii) culture of lymph node aspirate positive for *M. tuberculosis* complex bacteria with or without prior Ziehl Neelsen (ZN) or Auramine acid fast bacilli (AFB) positive smear. The patients were anti-tuberculous drug naive and had failed to respond to conventional antibiotics. Participants who consented to HIV testing were screened for antibodies against HIV 1 and HIV 2 by enzyme-linked immunosorbent assay (ELISA) after pre-test counseling. Patients were excluded if they had a neoplastic condition, culture negative lymph node aspirates or did not consent to fine needle aspiration (FNA).

After capturing the demographic data such as age, sex and HIV status, the participants’ relevant clinical history suggestive of TB was recorded. They were examined and in particular assessed for clinical, radiological and pathological parameters including lymph node site, size and consistence.

*Abdominal ultrasound scans* were performed to look for abnormal findings suggestive of abdominal TB with a Medison SonoAce 9900 ultrasound machine (Seoul, Korea) using a 7.5mHz convex probe and a 7.5 mHz linear probe. The presence of abdominal lymphadenopathy was considered significant if there were more than three lymph nodes or at least one lymph node 12 mm or more in size. Lymph nodes were sought in the paraaortic region, the porta hepatis region, the mesentery and the splenic hilum. The role of abdominal ultrasound in diagnosis of extrapulmonary or disseminated TB was previously evaluated, and a significant correlation was found between abdominal lymphadenopathy and active TB as diagnosed by smear or culture [23], thus abdominal ultrasound was found valuable in diagnosing EPTB [24].

*Chest x-ray* was performed with patients in standing posture and holding their breath, using a Schimadzu machine (Shimadzu-Japan), with the patient’s chest positioned 150 cm away from the x-ray tube. The chest X-ray was considered abnormal when it showed any of the following: lobar/segmental consolidation, cavitation, fibronodular lesions, pleural effusion, hilar and mediastinal lymph nodes, linear interstitial disease or miliary disease.

### Fine needle aspiration (FNA)

FNA was performed on consenting participants fulfilling inclusion criteria. Under aseptic conditions, the lymph node was immobilized with left hand to entirely access it while the right hand was used to aspirate. Using a 23G cutting needle and a 2 ml syringe lymph node aspiration was done with constant suction in a fan like fashion until the material appeared in the hub. The suction was then released, the needle withdrawn and the patient was asked to apply pressure to the punctured wound to prevent bleeding. Lymph node aspirates were cytologically analyzed and cultured for mycobacteria.

#### Cytomorphology

Aspirated lymph node material was expressed on to a glass slide with 3 ml of syringe air. The material was gently spread on the second slide by pulling the two slides facing each other gently apart. For each aspirate four slides were prepared, one for Papanicolaou stain, two for acid fast bacilli (AFB) stain (Ziehl Neelsen and fluorochrome respectively) while one was air dried for Romanowski stain (Biostains, UK) using standard protocols [25,26]. Cytomorphological findings were recorded in terms of presence or absence of granuloma formation, necrosis, Langhans giant cells and epitheloid transformed macrophages [27].

### Identification of the Uganda genotype strains

#### Sample processing for culture

Five ml of lymph node aspirate in distilled water was homogenized by digestion and decontaminated using the standard N-acetyl-L-cysteine (NALC)-Sodium hydroxide/Sodium citrate method [28] and the resulting sediment used for making smears and cultures on solid Lowenstein Jensen medium and the BACTEC MGIT 960 (Becton Dickinson Diagnostic Systems, Sparks, Md). Positive cultures were subjected to AFB staining for confirmation of mycobacterial growth.

DNA was extracted from growth using standard protocols [29,30] (Reagents from Sigma life Science, USA). Capilia TB assay (TAUN, Numazu, Japan), based on lateral flow immunochromatographic detection of a protein which is highly specific for MTB complex(MPB64) was used to differentiate *M. tuberculosis* complex isolates from non-tuberculous mycobacteria [31].

#### Spoligotyping

All *M. tuberculosis* complex strains were assayed by spoligotyping using standard protocols [32] (reagents from Ocimum Biosolution). Isolates were assigned specific spoligotype signatures (SIT) according to SITVITWEB database [33], which were used to determine their specific genotypes. The T2 lineage, to which the Uganda genotype belongs was identified. T2 lineage strains are characterized by a spoligotype pattern lacking spacers 40, or both 40 and 43 as previously described [34].

#### Region of difference (RD) analysis

This PCR based *M. tuberculosis* complex typing method depends on chromosomal region of difference (RD) deletion loci. The patterns of amplification products are visualized by agarose gel electrophoresis. All isolates were analyzed for a deletion at the RD724 locus, which is specific for the Uganda genotype as previously described [14].

For statistical analyses, genotypes were grouped into two: Uganda genotype (T2 strains with a deletion at the RD724 locus) and non-Uganda genotype (all other strain types in the collection).

### Statistical analyses

Clinical and socio-demographic data earlier collected were entered into Epidata v 3.1 and exported to SPSS v21.

Descriptive analysis was used for the baseline characteristics. Pearson chi square was used to measure differences between the two *M. tuberculosis* genotype categories with respect to demographic parameters. The independent *t*-test was used to compare the means of the continuous variables, age and lymph node size. Crude and multivariable logistic regression analysis was used to explore clinical, radiological and pathological features of the Uganda genotype compared with non-Uganda genotypes of *M. tuberculosis* controlling for potential confounding factors including sex, age and HIV status. Odds ratios (ORs) with corresponding 95% confidence intervals (CI) were calculated and are presented. A P value of <0.05 was considered evidence of significant difference.

## Results

Of the 283 patients with superficial lymphadenopathy and clinical features of TB and with cytological features consistent with tuberculous lymphadenitis enrolled, 121 (43%) had lymph node aspirates that turned out culture positive for *M. tuberculosis* complex, of which 55 (45%) were also ZN smear positive. 29 (53%) of these were Uganda genotype. Three (1.8%) samples were positive for non-tuberculous mycobacteria, while 159 (56%) turned out culture negative and were all excluded from further analysis.

### Patient characteristics

Of the 121 patients with tuberculous lymphadenitis 62 (52%) were female and 58 (48%) male, mean age being 28.5 years (range 2–61 years) (Table 1). The gender of one patient was not recorded. Of 114 patients who consented to HIV testing 75 (66%) were HIV positive while 39 (34%) were HIV negative. Seven patients did not consent to HIV testing. The major clinical signs were: a history of evening fever (all patients), night sweats (n = 98, 81%) and cough (n = 57, 47%). Sixty-six (55%) patients presented with lymphadenitis at a single site, and of those 25 (38%) had no signs of pulmonary and/or abdominal disease. Seventy-five (62%) patients were found to have pulmonary and/or abdominal involvement in addition to superficial lymphadenopathy.

**Table 1:** Baseline characteristics of patients infected with Uganda and non-Uganda strains

	**Uganda genotype n = 56 (46%)**	**Non-Uganda genotype n = 65 (54%)**	**P-Value**
**HIV Status**			
HIV +	31 (61%)	44 (70%)	0.311
HIV -	20 (39%)	19 (30%)	
**Age**			
Mean	27 (SD 10)	30 (SD 11)	0.225
**Sex**:			
Female	30 (55%)	32 (49%)	0.562
Male	25 (45%)	33 (51%)	

Chi-square and *t*-test.

The lymph node site distribution among the patients were as follows: right posterior cervical triangle 21 (17.4%), right anterior cervical triangle 10 (8.3%), left posterior cervical triangle 28 (23.1%), left anterior cervical triangle 3 (2.5%), left supra clavicular 2 (1.7%), right supra clavicular 1 (0.8%), left axillary 1 (0.8%), generalized superficial lymphadenopathy 16 (13.2%) and bilateral cervical lymphadenopathy 39 (32.2%).

The mean lymph node sizes calculated using the independent samples t- test model were 3.8 cm (standard deviation, SD 1.8) for patients infected with non-Uganda genotypes and 3.4 cm (SD 2.0) for patients infected with Uganda genotype (p = 0.305).

#### Cytomorphological features

**Figure 1:**
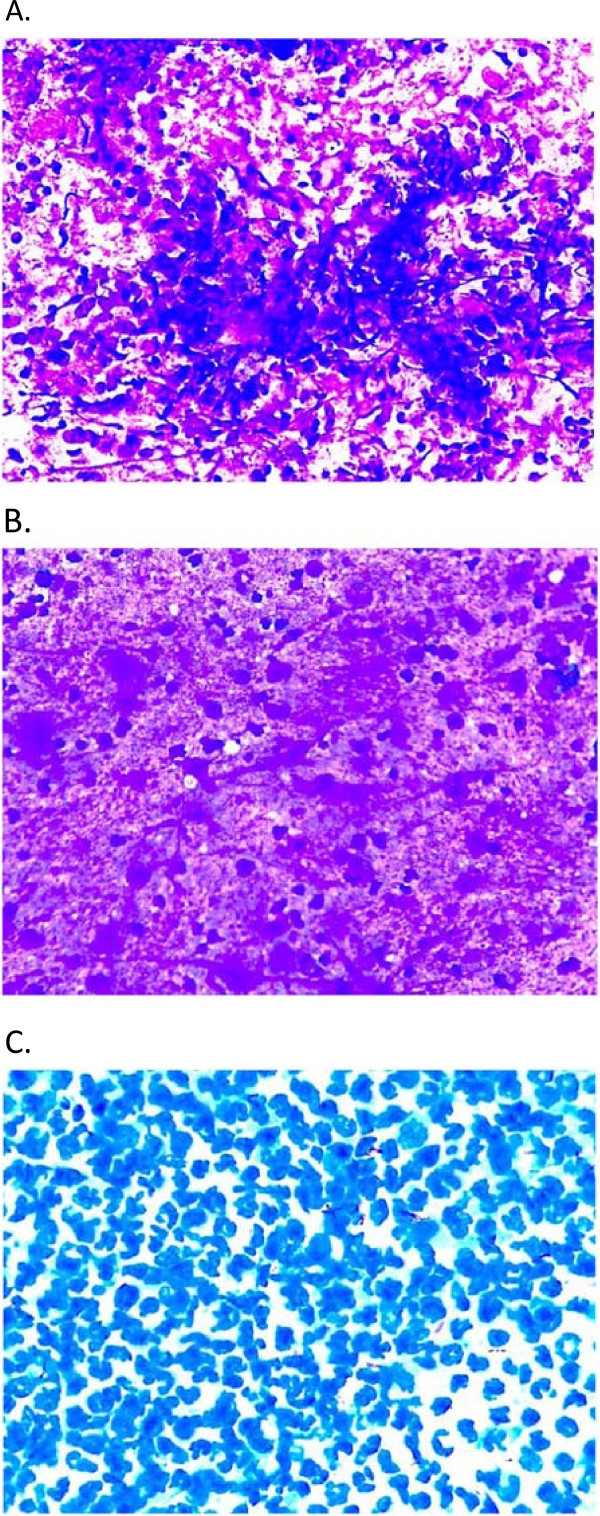
**Cytomorphological features of tuberculous lymphadenitis. (A)** Tuberculous lymphadenitis with chronic granulomatous inflammation comprising epitheloid cells, lymphocytes, multinucleated giant cells and caseous necrosis. **(B)** Tuberculous lymphadenitis with amorphous necrosis containing a few lymphocytes. **(C)** Tuberculous lymphadenitis with suppurative necrosis and a number of AFBs.

In practically all FNA samples (n = 113) there was necrosis (amorphous, caseous or suppurative). Granuloma were found in 93 (77%), Langhans giant cells in 53 (44%) and macrophages in 111 (92%) cases (Figure 1).

#### Abdominal ultrasound

Sixty patients had abdominal lymphadenopathy, ie more than three lymph nodes, or at least one lymph node of more than 12 mm in size. The involved anatomic sites included the para-aortic region, the porta hepatis region, the mesentery and the splenic hilum.

#### Chest X-ray

Forty-seven patients had an abnormal chest X-ray, with 6 having lobar/segmental consolidation, 2 cavitation, 4 fibronodular lesions, 7 pleural effusion, 12 hilar and mediastinal lymph nodes, 7 linear interstitial disease, and 9 miliary disease.

### *M. tuberculosis* complex genotypes

Spoligotyping revealed 54 distinct spoligotype patterns, of which 33 (61%) patterns were available in the SITVITWEB and 21 (39%) patterns were not found in the data base. Spoligotypes representing the Euro American lineage (to which the Uganda genotype belongs) were predominant (62%), followed by the Central Asian Strain (CAS) lineage (18%). There were three Beijing strains and no *Mycobacterium bovis*.

On characterization by spoligotyping and RD724 deletion analysis, the 121 *M. tuberculosis* complex isolates yielded 56 (46%) isolates of the Uganda genotype while 65 (54%) were non- Uganda genotypes.

### Association between patient characteristics and Uganda genotype

There were no statistically significant differences between patients infected with Uganda genotype and patients infected with non-Uganda genotypes with respect to the baseline parameters age, sex and HIV status (Table 1).

To investigate if there were differences in clinico-pathological characteristics of the tuberculous lymphadenitis patients infected with Uganda genotype versus those infected with other genotypes, we assessed the clinical, radiological and pathological features of patients in the two groups (Table 2). Since the disease outcome following infection with *M. tuberculosis* may be influenced by confounding factors like age, sex and HIV, multivariable logistic regression was used with *M. tuberculosis* genotype, age, sex and HIV status entered into the model to adjust for these confounding variables.

**Table 2:** **Clinical and immunopathological parameters of patients infected with Uganda genotype and those infected by non-Uganda genotypes of *M. tuberculosis***

**Patient characteristics**	**Uganda/Non-Uganda n (%)**	**Crude**		Adjusted*	
		**OR (95% CI)**	***P*-value**	**OR (95% CI)**	***P*-value**
*Clinical presentations*					
Fever less than 6 months	39 (70%)/44 (69%)	1.0 (0.5-2.3)	0.916	1.1 (0.5-2.5)	0.870
Night sweats	43 (77%)/55 (86%)	0.5 (0.2-1.4)	0.200	0.5 (0.2-1.4)	0.213
Cough	27 (48%)/30 (46%)	1.1 (0.5-2.2)	0.821	1.4 (0.7-3.1)	0.435
*Radiological Presentations*					
Abnormal Chest X-ray	20 (43%)/27 (47%)	0.8 (0.4-1.8)	0.624	0.8 (0.4-1.9)	0.644
Abdominal lymphadenopathy	21 (38%)/39 (60%)	0.4 (0.2-0.8)	0.014	0.5 (0.2-1.0)	0.046
*Lymph node Pathology*					
Single site lymphadenitis	34 (62%)/32 (50%)	1.6 (0.8-3.4)	0.197	1.4 (0.6-2.9)	0.435
Generalized lymphadenopathy	5 (9%)/11 (17%)	0.5 (0.2-1.5)	0.204	0.7 (0.2-2.1)	0.474
Lymph node ≥ 4 cm	15 (28%)/28 (45%)	0.5 (0.2-1.0)	0.055	0.6 (0.3-1.3)	0.157
Necrosis	52 (93%)/61 (94%)	0.9 (0.2-3.6)	0.827	0.8 (0.2-3.4)	0.741
Giant cells	28 (50%)/25 (38%)	1.6 (0.8-3.3)	0.203	1.3 (0.6-2.8)	0.569
Macrophages	53 (95%)/58 (89%)	2.1 (0.5-8.7)	0.290	1.5 (0.4-6.9)	0.577
Granulomas	47 (84%)/46 (71%)	2.2 (0.9-5.3)	0.091	1.9 (0.8-4.8)	0.196
Ziehl Neelsen positive smear	29 (52%)/42 (65%)	0.6 (0.3-1.2)	0.152	0.7 (0.3-1.5)	0.348

Adjusted* = Adjusted for Age, Sex and HIV status; OR = Odds ratio, Cl = Confidence Interval.

Logistic regression.

There were no statistically significant differences between the two groups (Uganda versus non-Uganda) with respect to cough, fever duration (more or less than 6 months), night sweats, lymph node size, and chest X ray findings (Table 3). However, patients infected with Uganda strains were significantly less prone to have abdominal lymphadenopathy compared to those infected with non-Uganda strains (OR 0.4, 95%CI 0.2-0.4,p = 0.014), even after adjusting for HIV infection, age and sex (OR = 0.5,95% CI 0.2-1.0,P = 0.046). Of the 16 patients with generalized lymphadenopathy 5 (31%) were infected with Uganda genotype strains while 11 (69%) were infected with non-Uganda genotypes.

**Table 3:** Distribution of the chest abnormalities among the patients infected with Uganda genotype and non-Uganda genotypes

**Chest X-ray abnormalities**	**Number**	**Uganda genotype**	**Non-Uganda genotypes**
Lobar/segmental consolidation	6 (12.8%)	5 (83%)	1 (17%)
Cavitation	2 (4.3%)	0 (0%)	2 (100%)
Fibro nodular lesions	4 (8.5%)	2 (50%)	2 (50%)
Pleural effusion	7 (14.9%)	2 (29%)	5 (71%)
Hilar and Mediastinal lymph nodes	12 (25.5%)	5 (42%)	7 (58%)
Linear interstitial disease	7 (14.9%)	1 (14%)	6 (86%)
Miliary disease*	9* (19.1%)	5 (56%)	4 (44%)

*7 patients are HIV positive, while the remaining 2 patients’ sero-status was unknown.

### Association of abdominal lymph node enlargement with features indicative of TB severity or dissemination

Abdominal lymph node enlargement was significantly associated with abnormal chest X-ray findings (p = 0.027), There was no statistically significant association between abdominal lymph node enlargement and generalized lymphadenopathy, AFB smear positivity or absence of granulomas (Table 4).

**Table 4:** Association of abdominal lymph node enlargement with features indicative of TB dissemination (i.e. generalized lymphadenopathy, abnormal chest X-Ray, Acid fast bacilli (AFB) positivity and absence of granuloma)

	**Abdominal lymphadenopathy n = 60 (49.6%)**	**No-abdominal lymphadenopathy n = 61 (50.4%)**	**P-Value**
**Generalized lymphadenopathy:**			
Present	30 (57%)	23 (43%)	0.170
Absent	20 (39%)	19 (30%)	
**Granuloma:**			
Present	46 (50%)	47 (50%)	0.960
Absent	14 (50%)	14(50%)	
**Abnormal findings on Chest X-ray:**			
Present	30 (64%)	17 (36%)	0.027
Absent	24 (41%)	34 (59%)	
**AFB smear positivity:** (Ziehl Neelsen or fluorochrome stain)			
Positive	39 (65%)	29 (47%)	0.053
Negative	21 (35%)	32 (53%)	

Chi-square test.

## Discussion

In this cross-sectional study of patients presenting with tuberculous lymphadenitis and clinical signs of TB we found a high prevalence of infection at other sites, with 9 patients with miliary pulmonary TB at the extreme end of the disease spectrum, while only 38% of the patients had single lymphadenitis with no abdominal or pulmonary engagement.

Lymphadenopathy (superficial as well as systemic, including abdominal lymphadenopathy) is regarded as a manifestation of EPTB, where the mode of infection includes haematogenous spread from a primary focus or in miliary TB, lymphatic spread from infected lymph nodes, ingestion of bacilli and direct spread from adjacent viscera [35]. There are indications that the homeostatic immune response in tuberculous lymphadenitis is fundamentally different from that of pulmonary TB [36].

Tuberculous lymphadenitis patients infected with Uganda genotype strains were significantly less prone to present with abdominal lymphadenopathy (p = 0.014) than those patients infected with non-Uganda strains, even after adjusting for confounding factors including age, sex and HIV co-infection (p = 0.046). Abdominal lymph node enlargement was itself significantly associated with abnormal chest X-ray findings (p = 0.027), one of the features indicative of TB severity or dissemination. Interestingly, the Uganda genotype which is responsible for close to 70% of pulmonary TB disease in our setting [14], in this study caused only 46% of the 121 cases of tuberculous lymphadenitis. Tuberculous lymphadenitis is the commonest form of extra pulmonary TB in high endemic areas [18,37]. Previous evidence also suggest that strains of the Euro-American lineage of *M. tuberculosis* to which *M. tuberculosis* Uganda genotype belongs less frequently cause EPTB [11,12].

Thus the Uganda genotype appears to have a reduced potential to disseminate and cause extra-pulmonary disease, and may inversely have a higher potential to cause pulmonary TB. Since pulmonary TB is more contagious than EPTB the Uganda genotype may have an advantage in transmission of TB which is reflected in the high incidence of this genotype in Uganda and surrounding areas [38]. The dominance of the Uganda genotype has however not been associated with pulmonary cavitation [39], which is associated with tissue liquafactive necrosis and cough generated aerosol, an efficient mechanism by which TB is spread.

A particular strain of Uganda genotype (SIT52) caused a large outbreak of TB in Sweden [40]. This strain exhibited extensive cell death of macrophages infected in vitro, with production of elevated amounts of tumor necrosis factor (TNF) compared to the virulent laboratory H37Rv strain [41]. The interplay of TNF and other cytokines in the early innate immune response is complex, but it is noteworthy that TNF neutralization in animal models including non-human primates resulted in disseminated disease in *M. tuberculosis* infected animals [42].

Risk factors for extra pulmonary TB include age, sex and HIV co-infection [21,43,44]. Not unexpectedly a high proportion (66%) of the patients in this study were HIV-infected, a figure which is similar to studies of patients with extra-pulmonary TB in other high endemic African countries [45,46]. It is well known that HIV co-infection represents an increased risk of dissemination of TB involving multiple organs, through several immunological mechanisms not yet quite well understood [47], and infection with HIV increases the incidence and severity of abdominal TB [37]. Thus 7 patients with miliary TB were HIV-infected while the sero status of the remaining 2 patients was not known. However, HIV-status did not affect the proportion of patients infected with Uganda genotype, and only marginally affected the negative correlation between Uganda genotype and abdominal lymphadenitis, indicating that HIV-status was not important for the differences in clinical manifestations between Uganda and non-Uganda genotypes.

The indication of a lower tendency to form extrapulmonary disease by the Uganda genotype compared with the other genotypes is in line with a recent large study of TB cases that showed a relationship between phylogenetic lineage and clinical site (pulmonary versus extra pulmonary) of TB [48]. In a study from Vietnam disease caused by the Euro-American lineage was significantly more likely to be pulmonary than meningeal, which suggests that this lineage is less capable of extrapulmonary dissemination in this population [49].

Extrapulmonary disease has been associated with the East Asian/Beijing genotype [50]. Patients infected with strains of Beijing genotype were found to have significantly lower frequency of fever and pulmonary cavitation than those infected with other genotypes of *M. tuberculosis*[51], and were associated with characteristic radiological patterns [52]. These associations seem to hold for some populations and not for others [53,54], and may be explained by the fact that the Beijing lineage has evolved into distinct branches defined by specific RD deletions [55] with different impact on the immune response [56]. On the other hand there was no difference in lineage distribution between cases of pulmonary and extrapulmonary TB in a study from Ethiopia [57], a country with a high incidence of tuberculous cervical lymphadenitis [57], and a study from South Africa [58] found no significant relationship between mycobacterial genotype and EPTB. Yet, it is increasingly evident that the genetic diversity of *M. tuberculosis* strains contributes to the wide clinical spectrum of TB [59,60].

The limitation of this study is the fact that unlike animal models, the patients live in a heterogeneous socio-economic environment which may confer varying susceptibility and resistance to TB. Factors like clinical stages of TB and/or HIV and immunosuppressive states like malnutrition and diabetes mellitus may contribute to disease outcome in TB patients.

## Conclusion

In summary, tuberculous lymphadenitis patients infected with strains of Uganda genotype were significantly less prone to have abdominal lymphadenopathy and may be less susceptible to developing severe or disseminated TB. The frequency of Uganda genotype in this study was lower than the frequency of Uganda genotype reported for pulmonary TB in the same setting. Genotyping of *M. tuberculosis* may aid clinicians make better therapeutic decisions and predict patient prognosis.

Further studies are indicated to elucidate the clinical-pathological significance of the genetic diversity of *M. tuberculosis* strains and lineages in Uganda for understanding the outcome of infection with the Uganda genotype.

## Abbreviations

TB: Tuberculosis; HIV: Human immunodeficiency virus; ZN: Ziehl-Neelsen; M.tb: Mycobacteria tuberculosis; MOTT: Mycobacteria other than tuberculosis; MGIT: Mycobacteria other than tuberculosis; FNA: Fine needle aspiration; DQ: Diff. quick stain; Pap: Papanicolaou stain; OR: Odds ratio; CI: Confidence interval.

## Competing interests

The authors declare that they have no competing interests.

## Authors’ contributions

DW: Participated in the research conceptual development, drafting of manuscript, data acquisition, analysis and interpretation, and to intellectual content. EK: Participated in molecular biology studies and made substantial contribution to interpretation of data and intellectual discussion. GM: Participated in microbiology studies and contributed to intellectual content. GK: Participated in the conceptual development,drafting and critically revising the manuscript. Contributed substantially to the intellectual content and gave the final approval of the manuscript. BA: Participated in the research conceptual development, drafting and revising the manuscript and to the intellectual content. MJ: Participated in the research conceptual development and design and contributed to the intellectual content of the manuscript. All authors read and approved the final manuscript.

## Pre-publication history

The pre-publication history for this paper can be accessed here:

http://www.biomedcentral.com/1472-6890/14/14/prepub
